# Electrocaloric effect in ferroelectric nanowires from atomistic simulations

**DOI:** 10.1038/srep17294

**Published:** 2015-11-27

**Authors:** R. Herchig, C.-M. Chang, B. K. Mani, I. Ponomareva

**Affiliations:** 1 Department of Physics, University of South Florida, Tampa, Florida 33620, USA; 2 Cyber-Enabled Research, Michigan State University, Biomedical & Physical Sciences Building, 567 Wilson Road, Room 1440, East Lansing, Michigan 48824-1226

## Abstract

Electrocaloric effect is presently under active investigation owing to both the recent discoveries of giant electrocaloric effects and its potential for solid state cooling applications. We use first-principles-based direct simulations to predict the electrocaloric temperature change in ferroelectric ultrathin nanowires. Our findings suggest that in nanowires with axial polarization direction the maximum electrocaloric response is reduced when compared to bulk, while the room temperature electrocaloric properties can be enhanced by tuning the ferroelectric transition temperature. The potential of ferroelectric nanowires for electrocaloric cooling applications is discussed.

Ferroelectrics have received much attention in the recent years as potential candidates for solid state cooling applications^1^,^2^,^3^,^4^. Such applications take advantage of the electrocaloric effect (ECE) that is defined as a reversible change in temperature under the adiabatic application of an electric field. The interest in ferroelectrics as potential electrocaloric materials is largely due to the findings of giant ECE in several ferroics^5^,^6^,^7^,^8^. Subsequent studies revealed that ferroelectric electrocalorics might have other prospects, thanks to their potential to demonstrate multiple caloric effects^9^,^10^,^11^. Some examples include the coexistence of electrocaloric and piezocaloric effects in the same material^10^,^11^ as well as the coexistence of positive and negative ECE in the same ferroelectric below its Curie temperature^9^. From a practical point of view ferroelectric nanostructures appear to be most attractive as they allow the application of much larger electric fields^1^,^5^. Indeed, majority of giant electrocaloric effects were reported for thin film samples^5^,^6^,^8^,^12^. Interestingly, while ferroelectric thin films received much attention in the caloric effect studies, the research on other low-dimensional structures, such as nanowires and nanodots, is very limited^13^,^14^,^15^. Very large positive and negative ECE was predicted in PbTiO_3_ nanoparticles with a vortex domain structure^13^. Ref. 14 predicted giant ECE in ferroelectric nanotubes that originates from an unusual domain transition. Given the very limited number of studies, it is presently unclear what effect the reduced dimensionality has on the electrocaloric properties of ferroelectrics.

The aims of this Paper are: (i) to predict the intrinsic features of ECE in ferroelectric ultrathin nanowires; (ii) to elucidate the effect of reduced dimensionality and size on the electrocaloric properties of nanoscale ferroelectrics; (iii) to explore the potential of ferroelectric nanowires for nanoscale cooling applications.

We simulate three nanowires made of PbTiO_3_, BaTiO_3_ and KNbO_3_ ferroelectric perovskites. These materials are chosen as representatives of ferroelectrics with single (PbTiO_3_) and multiple (BaTiO_3_ and KNbO_3_) phase transitions. Bulk PbTiO_3_ undergoes a single transition from a paraelectric cubic to a ferroelectric tetragonal phase at 763 K^16^. Bulk BaTiO_3_ undergoes a sequence of phase transitions starting from paraelectric cubic to a ferroelectric tetragonal phase transition at 393 K, followed by a transition to a ferroelectric orthorhombic phase at 273 K, and a transition to a ferroelectric rhombohedral phase at 183 K^17^. Bulk KNbO_3_ exhibits the same sequence of phase transitions as BaTiO_3_, but at elevated temperatures of 708 K (the Curie point), 498 K, and 263 K^18^. Ferroelectric perovskites have been successfully synthesized in quasi-one-dimensional forms^19^. These include BaTiO_3_ nanowires^20^, PbTiO_3_ nanorods^21^ and rodlike nanocrystalline KNbO_3_^22^. Here we model nanowires with a 12 × 12 unit cells square cross section grown along the <001> pseudocubic direction. Each nanowire is simulated using a 12 × 12 × 20 supercell which is periodic along the nanowire’s axial direction (*z*− Cartesian axis in our case). Such setup models a nanowire with the aspect ratio *D*/*L* ≪ 1, where *D* and *L* are the nanowire’s diameter and length, respectively. Note that some of our results were cross-checked using 12 × 12 × 24 supercell. No dependence on the supercell size along the periodic direction was found. The energy of the supercell is given by the first-principles effective Hamiltonian^23^. The degrees of freedom for the Hamiltonian include local modes, **u**_*i*_, that are proportional to the dipole moment in the unit cell, and strain variables tensors *η*_*i*_ (in Voigt notations) that are responsible for mechanical deformations of a unit cell. The energy of the Hamiltonian is^23^


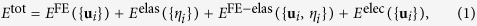


where *E*^FE^ is the energy associated with the ferroelectric local modes and includes contributions from the dipole-dipole interactions, short-range interactions, and on-site self energy as defined in ref. 23. The on-site self energy gives the energy of an isolated local mode with respect to the perfect cubic structure and contains harmonic as well as anharmonic contributions. The second term, *E*^elas^, is the elastic energy associated with the unit cell deformations. *E*^FE−elas^ is the energy contribution due to the interactions between the ferroelectric local modes and the strain. The last term, 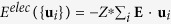, where Z^*^ is the Born effective charge, gives the interaction energy between the local modes and an external electric field, **E**. The first-principles parameters for PbTiO_3_ are those from ref. 24. Parameters for BaTiO_3_ and KNbO_3_ were computed from first-principles and and are given in Table 1. The Hamiltonian of Eq.(1) correctly reproduces most of the thermodynamical properties of bulk PbTiO_3_, BaTiO_3_ and KNbO_3_. In particular, the PbTiO_3_ Hamiltonian predicts a single transition from a paraelectric cubic to a ferroelectric tetragonal phase at 605 K. The present parametrization of BaTiO_3_ correctly reproduces the sequence of phase transitions in this material with a transition to a ferroelectric tetragonal phase at 405 K, to a ferroelectric orthorhombic phase at 260 K, and to a ferroelectric rhombohedral phase at 195 K. Similarly, the present parametrization of KNbO_3_ predicts the correct sequence of phase transition in bulk KNbO_3_ with computational transition temperatures of 705, 430, and 345 K for the three phase transitions. Note, that the long-range dipole-dipole interactions in nanowires are computed using the approach of ref. 25.

The surface of the nanowire creates a boundary at which the polarization discontinuity may occur. Such discontinuity could be eliminated (fully or partially) by an intrinsic or extrinsic free charge - the compensating charge. Here we simulate a realistic situation of a partial surface charge compensation by a limited number of free carriers. Technically this is achieved by compensating only 10% of the surface charge (open-circuit electrical boundary conditions) using the approach of ref. 25. Such setup models experimentally realizable conditions^26^,^27^. Under open-circuit electrical boundary conditions nanowires do not develop polarization along their truncated dimensions as it would result in a large residual depolarizing field^19^,^27^,^28^,^29^,^30^,^31^,^32^,^33^. The energy given by the effective Hamiltonian is used in the framework of classical Monte Carlo (MC) and Molecular Dynamics (MD) to compute finite temperature properties of the nanowires. Similar computational approach has been previously used to study various properties of ferroelectric nanowires^28^,^34^,^35^.

We begin by investigating the sequence of phase transitions in nanowires using the simulated annealing approach. In such an approach the simulations begin at temperature much above the Curie point and proceeds in steps of 5 K decrements until the simulated temperature reaches 5 K. For each temperature 3*10^5^ MD steps are used. The temperature evolution of the polarization obtained from the annealing simulations is given in Fig. 1. For comparison we also include data for bulk. Note that the bulk material is simulated by applying periodic boundary conditions along all three Cartesian directions. Figure 1(a) shows the data for PbTiO_3_ nanowire and the bulk. It demonstrates that the reduction in dimensionality leads to a decrease in transition temperature and a smearing of the phase transition^36^. The decrease in transition temperature is in a qualitative agreement with the predictions from the semi-phenomenological theory^37^,^38^,^39^. For BaTiO_3_ nanowire (see Fig. 1(b)) we find very little change in the ferroelectric transition temperature as compared to the bulk. However, the phase transition sequence is drastically different from the one in the bulk. We do not find any polarization along the nanowire’s truncated dimensions due to a prohibitively large depolarizing field associated with the chosen electrical boundary conditions. The ferroelectric transition in the nanowire has a slightly smeared character as compared to bulk. KNbO_3_ nanowire data are given in Fig. 1(c) and exhibit trends similar to the BaTiO_3_ nanowire. In particular, the ferroelectric phase transition appears to be smeared, while no polarization is developed along the nanowire’s truncated dimensions.

To gain further insight into the ferroelectric phases and phase transitions character we computed dielectric constant *ε*_33_ for all nanowires and their bulk counterparts using a direct simulation approach. In such an approach the electric field is applied along the *z*– Cartesian direction in increments of 36 kV/cm, while the polarization is computed for each value of the electric field. For each electric field increment 5*10^5^ MD steps are used. The zero field slope in the polarization versus electric field data is used to calculate the dielectric constant. The data are given in Fig. 1(d–f). For PbTiO_3_ nanowire we observe a smearing of the dielectric constant. Similar trends were also observed for the ferroelectric thin films^40^. For the BaTiO_3_ nanowire the smearing of the dielectric constant is less pronounced. In this case we notice a small peak in the dielectric constant around 120 K. To trace the origin of the peak we turn to the dipole pattern evolution which reveals that at 135 K the nanowire undergoes a transition into a polydomain phase that is best described by a combination of two order parameters - the polarization and the toroidal moment of polarization^41^, **G**. The toroidal moment of polarization is defined as 
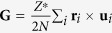
, where *N* is the number of unit cells and **r**_*i*_ is the location of the local mode *i*^41^. In this case both order parameters align along nanowire’s axial direction. The temperature evolution of the toroidal moment of polarization is given in the inset to Fig. 1(e). A similar transition occurs in KNbO_3_ nanowire at a higher temperature of 420 K. The dielectric constant of KNbO_3_ nanowire is also smeared.

Our computational data predict that ferroelectric nanowires with a poorly compensated surface charge develop ferroelectric phases with a polarization along the axial direction. Nanowires may develop polydomain phases with multiple order parameters. The common features are the smearing of the phase transition and the dielectric constant. They can be attributed to the decrease in the correlation length due to the reduced dimensionality. Previously, the correlation effects were found to decrease the transition temperature in nanorods^38^. Smaller correlation lengths are usually associated with diffuse phase transitions^42^. Interestingly, ferroics with smeared phase transitions are quite desirable for caloric applications as they usually demonstrate a broadened caloric response^43^.

Having established the equilibrium phases and electric properties of nanowires, we turn to the modeling of the ECE. To simulate the ECE we apply an electric field along the nanowire’s axial direction under adiabatic conditions using the computational approach of refs 9,10. In bulk the electric field is applied along the polarization direction. Technically, the electric field was first applied and then removed very slowly at a rate of 100 V/m per one MC sweep to ensure reversibility. The electrocaloric temperature was computed as a function of the applied field. The electrocaloric change in temperature as a function of the initial temperature is given in Fig. 2 for a few different values of the applied electric field. We have also included data for the linear electrocaloric response, *dT*/*dE*, at low fields which were computed by taking the zero field slope of temperature versus electric field data. It should be noted that our computational data for BaTiO_3_ are in excellent agreement with the direct experimental measurements on BaTiO_3_ multilayer thick film (see Fig. 2(b)). We begin the discussion with the PbTiO_3_ nanowire. Here we find a reduction in the maximum electrocaloric response as compared to the bulk. Such a reduction could be elucidated with the help of Maxwell relation for the electrocaloric change in temperature 
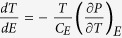
, where *C*_*E*_ is the volumetric heat capacity, *P* is the polarization, *T* and *E* are the temperature and the electric field, respectively. For low fields the relationship can be approximated as 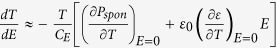, where *P*_*spon*_ is the spontaneous polarization and *ε* is the dielectric constant. The latter expression suggests that the linear ECE is proportional to the pyroelectric coefficient 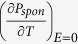 and the derivative of the dielectric constant with respect to temperature. Both the spontaneous polarization and the dielectric constant are reduced in the nanowire as evident from Fig. 1 due to the reduced correlation length. This explains the observed decrease in the maximum ECE in the nanowire as compared to bulk. Similarly, we observe a reduced maximum electrocaloric response in both BaTiO_3_ and KNbO_3_ nanowires as compared to their bulk counterparts. Both BaTiO_3_ and KNbO_3_ mostly loose their electrocaloric properties in polydomain phases. To elucidate the reason for this we first notice that below the ferroelectric transition temperature the ECE is dominated by the pyroelectric coefficient. Indeed in a ferroelectric phase the two terms in the Maxwell relation have the opposite sign (as could be seen from inspection of Fig. 1) and compete with each other. The overall sign of the ECE is determined by the largest of the two terms and is positive in this case (see Fig. 2). The positive sign of ECE implies that the pyroelectric coefficient 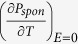 makes a dominant contribution to the ECE. In the polydomain phase the spontaneous polarization depends on the temperature only weakly (see Fig. 1) resulting in very small values of the pyroelectric coefficient and the associated ECE. More intuitively, the reduction of ECE in a polydomain phase could be understood by recalling that inside each domain the polarization is at an angle with the applied electric field. As a result the application of a relatively low electric field does not significantly affect the configurational disorder and the entropy associated with it. The lack of the entropy change results in a negligible ECE.

To investigate the potential of the nanowires for cooling applications we computed the relative cooling power^44^ *RCP* = Δ*T*_*max*_ × *δT*_*FWHM*_, where Δ*T*_*max*_ is the maximum of the electrocaloric temperature change and, *δT*_*FWHM*_ is the full width at half maximum. The *RCP* values for different electric fields are given in Table 2. In all cases we find that the *RCP* is reduced in nanowires, on an average by 20%.

Interestingly, our computational data predict that, while the maximum electrocaloric Δ*T* as well as *RCP* are reduced in nanowires, their room temperature response could be significantly enhanced. Indeed all nanowires exhibit a decrease in the ferroelectric transition temperature and associated shift in both dielectric and electrocaloric response (see Figs 1(c,d) and 2(c,d)). As a result the room temperature Δ*T* of BaTiO_3_ nanowire remains comparable to Δ*T* in bulk, while PbTiO_3_ nanowire exhibits a 30% enhancement in the room temperature Δ*T*, which is very attractive for near room temperature refrigeration.

In summary, we studied the ECE in poorly compensated ferroelectric ultrathin nanowires and compared the findings to the ECE in bulk ferroelectrics. The computational data demonstrate a reduction in the ECE in nanowires as compared to bulk which is attributed to the reduced correlation length. In nanowires with polydomains the ECE is nearly negligible. The electrocaloric change in temperature exhibits a strong correlation with the dielectric susceptibility. Among the three ferroelectric perovskites studied in this work we find the largest ECE and *RCP* in PbTiO_3_ followed by KNbO_3_ and BaTiO_3_. This behavior is well correlated with the trends in the dielectric susceptibility and the spontaneous polarization data where we find the largest values in PbTiO_3_, followed by KNbO_3_ and BaTiO_3_. While nanowires exhibit reduction in maximum ECE, they offer the opportunity to tune the electrocaloric temperature change through variation of the transition temperature. For example, in case of PbTiO_3_ nanowires we find nearly 30% increase in the room temperature Δ*T*, which makes such nanowires attractive for room temperature cooling applications.

## Additional Information

**How to cite this article**: Herchig, R. *et al.* Electrocaloric effect in ferroelectric nanowires from atomistic simulations. *Sci. Rep.* **5**, 17294; doi: 10.1038/srep17294 (2015).

## Figures and Tables

**Figure 1:**
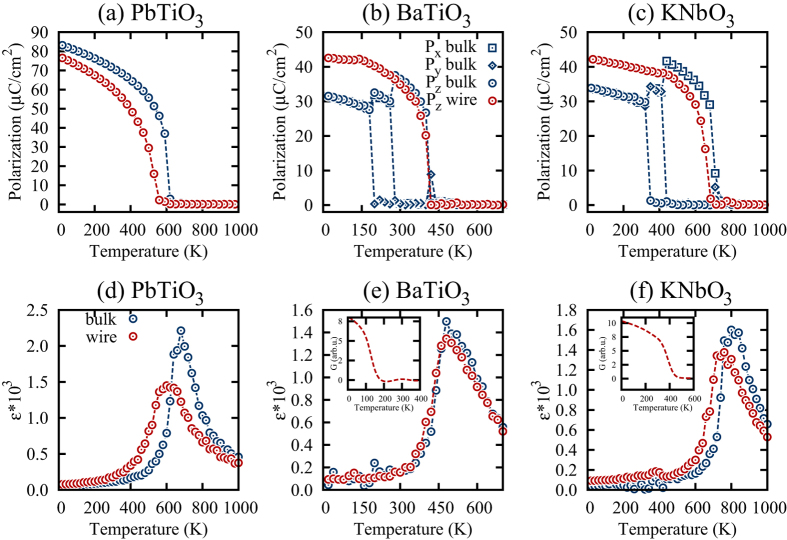
Dependence of the polarization components on the temperature in bulk and nanowires (**a**–**c**). Dependence of the dielectric constant on the temperature in bulk and nanowire (**d**–**f**). The insets give the toroidal moment of polarization.

**Figure 2:**
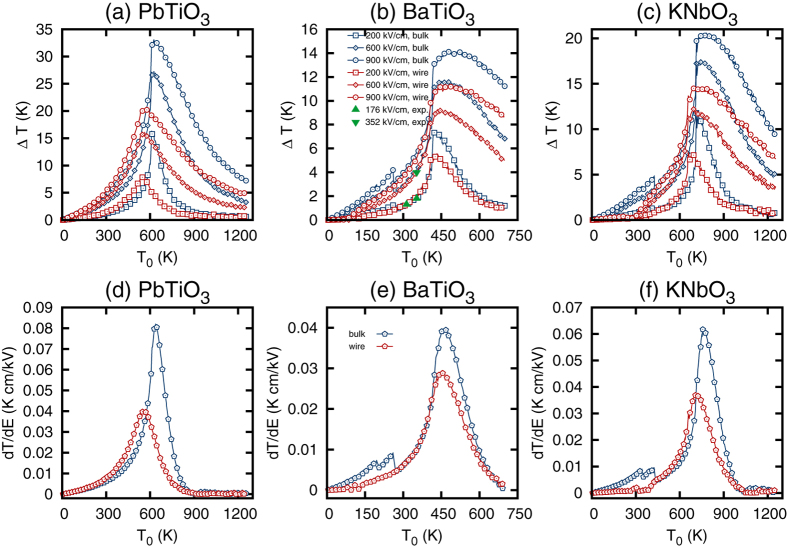
The electrocaloric change in temperature as a function of initial temperature for a few values of the electric field [(**a**–**c**)]. In (**b**) the triangles give the experimental data from the direct measurements on thick films^45^. The linear ECE as a function of the initial temperature. Note, that for bulk the electric field is applied along the polarization direction.

**Table 1:** First-principles parameters for BaTiO_3_ and KNbO_3_ in atomic units using the notations of ref. 23.

BaTiO_3_						
On-site	*κ*_2_	0.130175	*α*	0.224444	*γ*	−0.331164
Intersite	*j*_1_	−0.015605	*j*_2_	−0.0079159	*j*_5_	−0.0028348
	*j*_3_	0.005074	*j*_4_	−0.0046502		
	*j*_6_	0.002085	*j*_7_	0.0037973		
Elastic	*B*_11_	3.507579	*B*_12_	1.172548	*B*_44_	1.3057916
Coupling	*B*_1*xx*_	−1.557712	*B*_1*yy*_	−0.0065895	*B*_4*yz*_	−0.01217361
Other	Z^*^	12.336	*ε*_∞_	6.883		
**KNbO_3_**						
On-site	*κ*_2_	0.1735972	*α*	0.243023	*γ*	−0.415575
Intersite	*j*_1_	−0.01514268	*j*_2_	−0.03489560	*j*_5_	−0.01150196
	*j*_3_	0.00411942	*j*_4_	−0.002840009		
	*j*_6_	0.00128668	*j*_7_	0.000842484		
Elastic	*B*_11_	4.511186	*B*_12_	0.721808	*B*_44_	0.936883
Coupling	*B*_1*xx*_	−2.066661	*B*_1*yy*_	0.238860	*B*_4*yz*_	−0.035044
Other	Z^*^	11.829	*ε*_∞_	6.836		

Lattice constants are 7.4713 and 7.5505 a.u., respectively.

**Table 2:** *RCP* values in K^2^ for different electric fields.

Electric field (kV/cm)	PbTiO_3_		BaTiO_3_		KNbO_3_	
	nanowire	bulk	nanowire	bulk	nanowire	bulk
50	448	560	248	298	393	540
200	1628	1768	821	936	1253	1743
500	4514	5914	2593	3202	4040	4973

## References

[b1] Scott J. F. Electrocaloric materials. Ann. Rev. Mater. Sci. 41, 229 (2011).

[b2] Chukka R., Vandrangi S., Shannigrahi S. & Chen L. An electrocaloric device demonstrator for solid-state cooling. EPL-Europhys. Lett. 103 (2013).

[b3] Moya X., Kar-Narayan S. & Mathur N. D. Caloric materials near ferroic phase transitions. Nat. Mater. 13, 439–450 (2014). 24751772 10.1038/nmat3951

[b4] Valant M. Electrocaloric materials for future solid-state refrigeration technologies. Prog. Mater. Sci. 57, 980 (2012).

[b5] Mischenko A. S., Zhang Q., Scott J. F., Whatmore R. W. & Mathur N. D. Giant electrocaloric effect in thin-film PbZr_0.95_Ti_0.05_O_3_. Science 311, 1270–1271 (2006). 16513978 10.1126/science.1123811

[b6] Neese B. *et al.* Large electrocaloric effect in ferroelectric polymers near room temperature. Science 321, 821 (2008). 18687960 10.1126/science.1159655

[b7] Akcay G., Alpay S. P., Mantese J. V. & Rossetti G. A. Jr Magnitude of the intrinsic electrocaloric effect in ferroelectric perovskite thin films at high electric fields. Appl. Phys. Lett. 90, 252909 (2007).

[b8] Mischenko A. S., Zhang Q., Whatmore R. W., Scott J. F. & Mathur N. D. Giant electrocaloric effect in the thin film relaxor ferroelectric 0.9PbMg_1*/*3_Nb_2*/*3_O_3_–0.1PbTiO_3_ near room temperature. Appl. Phys. Lett. 89, 242912 (2006).

[b9] Ponomareva I. & Lisenkov S. Bridging the macroscopic and atomistic descriptions of the electrocaloric effect. Phys. Rev. Lett. 108, 167604 (2012). 22680758 10.1103/PhysRevLett.108.167604

[b10] Lisenkov S. & Ponomareva I. Giant elastocaloric effect in ferroelectric Ba_0.5_Sr_0.5_TiO_3_ alloys from first-principles. Phys. Rev. B 86, 104103 (2012).

[b11] Lisenkov S., Mani B. K., Chang C.-M., Almand J. & Ponomareva I. Multicaloric effect in ferroelectric PbTiO_3_ from first principles. Phys. Rev. B 87, 224101 (2013).

[b12] Peng B., Fan H. & Zhang Q. A giant electrocaloric effect in nanoscale antiferroelectric and ferroelectric phases coexisting in a relaxor Pb_0.8_Ba_0.2_ZrO_3_ thin film at room temperature. Adv. Funct. Mater. 23, 2987–2992 (2013).

[b13] Zeng Y. K. *et al.* Influence of vortex domain switching on the electrocaloric property of a ferroelectric nanoparticle. RSC Adv. 4, 30211 (2014).

[b14] Liu M. & Wang J. Giant electrocaloric effect in ferroelectric nanotubes near room temperature. Sci. Rep. 5 (2015). 10.1038/srep07728 PMC4289897 25578434

[b15] Prosandeev S., Ponomareva I. & Bellaiche L. Electrocaloric effect in bulk and low-dimensional ferroelectrics from first principles. Phys. Rev. B 78, 052103 (2008).

[b16] Shirane G., Axe J. D., Harada J. & Remeika J. P. Soft ferroelectric modes in lead titanate. Phys. Rev. B 2, 155–159 (1970).

[b17] Mason W. P. Piezoelectric Crystals and Their Application to Ultrasonics (D. Van nostrand Company, Inc., Toronto New York London, 1950).

[b18] Hewat A. W. Cubic-tetragonal-orthorhombic-rhombohedral ferroelectric transitions in perovskite potassium niobate: neutron powder profile refinement of the structures. J. Phys. C: Solid State 6, 2559–2572 (1973).

[b19] Rørvik P. M., Grande T. & Einarsrud M.-A. One-dimensional nanostructures of ferroelectric perovskites. Adv. Mater. 23, 4007–4034 (2011). 21796684 10.1002/adma.201004676

[b20] Urban J., Spanier J., Ouyang L., Yun W. & Park H. Single-crystalline barium titanate nanowires. Adv. Mater. 15, 423–426 (2003).

[b21] Deng Y. *et al.* Synthesis and characterization of single-crystal PbTiO_3_ nanorods. Mater. Lett. 59, 3272–3275 (2005).

[b22] Liu J.-F., Li X.-L. & Li Y.-D. Synthesis and characterization of nanocrystalline niobates. J. Cryst. Growth 247, 419–424 (2003).

[b23] Zhong W., Vanderbilt D. & Rabe K. First-principles theory of ferroelectric phase transitions for perovskites: The case of BaTiO_3_. Phys. Rev. B 52, 6301 (1995). 10.1103/physrevb.52.6301 9981860

[b24] Mani B. K., Chang C.-M. & Ponomareva I. Atomistic study of soft-mode dynamics in PbTiO_3_. Phys. Rev. B 88, 064306 (2013).

[b25] Ponomareva I., Naumov I. I., Kornev I., Fu H. & Bellaiche L. Atomistic treatment of depolarizing energy and field in ferroelectric nanostructures. Phys. Rev. B 72, 140102 (2005).

[b26] Highland M. J. *et al.* Equilibrium polarization of ultrathin PbTiO_3_ with surface compensation controlled by oxygen partial pressure. Phys. Rev. Lett. 107, 187602 (2011). 22107673 10.1103/PhysRevLett.107.187602

[b27] Wang Z., Suryavanshi A. P. & Yu M.-F. Ferroelectric and piezoelectric behaviors of individual single crystalline BaTiO_3_ nanowire under direct axial electric biasing. Appl. Phys. Lett. 89, 082903 (2006).

[b28] Ponomareva I., Naumov I. I. & Bellaiche L. Low-dimensional ferroelectrics under different electrical and mechanical boundary conditions: Atomistic simulations. Phys. Rev. B 72, 214118 (2005).

[b29] Pilania G., Alpay S. P. & Ramprasad R. *Ab initio* study of ferroelectricity in BaTiO_3_ nanowires. Phys. Rev. B 80, 014113 (2009).

[b30] Pilania G. & Ramprasad R. Complex polarization ordering in PbTiO_3_ nanowires: A first-principles computational study. Phys. Rev. B 82, 155442 (2010).

[b31] Sqterli R. *et al.* Polarization control in ferroelectric PbTiO_3_ nanorods. J. of Appl. Phys. 108, 124320 (2010).

[b32] Wang Z., Hu J. & Yu M.-F. One-dimensional ferroelectric monodomain formation in single crystalline BaTiO_3_ nanowire. Appl. Phys. Lett. 89, 263119 (2006).

[b33] Wang Z., Hu J. & Yu M.-F. Axial polarization switching in ferroelectric BaTiO_3_ nanowire. Nanotechnology 18, 235203 (2007).

[b34] Louis L. *et al.* Low-symmetry phases in ferroelectric nanowires. Nano Lett. 10, 1177–1183 (2010). 20230042 10.1021/nl9034708

[b35] McCash K., Srikanth A. & Ponomareva I. Competing polarization reversal mechanisms in ferroelectric nanowires. Phys. Rev. B 86, 214108 (2012).

[b36] Bratkovsky A. M. & Levanyuk A. P. Smearing of phase transition due to a surface effect or a bulk inhomogeneity in ferroelectric nanostructures. Phys. Rev. Lett. 94, 107601 (2005). 15783521 10.1103/PhysRevLett.94.107601

[b37] Huang H., Sun C. Q., Tianshu Z. & Hing P. Grain-size effect on ferroelectric PbZr_1*−x*_Ti_*x*_O_3_ solid solutions induced by surface bond contraction. Phys. Rev. B 63, 184112 (2001).

[b38] Morozovska A. N., Eliseev E. A. & Glinchuk M. D. Ferroelectricity enhancement in confined nanorods: Direct variational method. Phys. Rev. B 73, 214106 (2006).

[b39] Morozovska A. N., Glinchuk M. D. & Eliseev E. A. Phase transitions induced by confinement of ferroic nanoparticles. Phys. Rev. B 76, 014102 (2007).

[b40] Parker C. B., Maria J.-P. & Kingon A. I. Temperature and thickness dependent permittivity of (Ba, Sr)TiO_3_ thin films. Appl. Phys. Lett. 81, 340–342 (2002).

[b41] Naumov I. I., Bellaiche L. & Fu H. Unusual phase transitions in ferroelectric nanodisks and nanorods. Nature 432, 737 (2004). 15592408 10.1038/nature03107

[b42] Lines M. & Glass A. Principles and Applications of ferroelectrics and related materials (Clarendon Press-Oxford, 1977).

[b43] Li B. *et al.* Intrinsic electrocaloric effects in ferroelectric poly(vinylidene fluoride-trifluoroethylene) copolymers: Roles of order of phase transition and stresses. Appl. Phys. Lett. 96, 102903 (2010).

[b44] Gschneidner K. A. & Pecharsky V. K. Magnetocaloric materials. Annu. Rev. Mater. Sci. 30, 387–429 (2000).

[b45] Bai Y., Zheng G. & Shi S. Direct measurement of giant electrocaloric effect in BaTiO3 multilayer thick film structure beyond theoretical prediction. Appl. Phys. Lett. 96, 192902 (2010).

